# Artificially induced altitudes aerobic and anaerobic performance differences on double-poling ergometer in elite young cross-country skiers

**DOI:** 10.12688/f1000research.166884.1

**Published:** 2025-07-28

**Authors:** Peter Bartik, Jiří Suchý, Ladislav Pyšný, Jana Pyšná, Dragoş Ioan Tohănean, Pablo Prieto González, Peter Šagát

**Affiliations:** 1 Sport Sciences and Diagnostics Research Lab, Prince Sultan University, Riyadh, Saudi Arabia; 2 Faculty of Education, Charles University, Prague, Czechia, Czech Republic; 3 Department of Physical Education and Sport, Faculty of Education, J.E. Purkyne University in Ústí nad Labem, Ústí nad Labem Region, Czech Republic; 4 Faculty of Physical Education and Mountain Sports, Transilvania University of Brașov, Brasov, Romania

**Keywords:** cross-country skiing; double-poling; artificial hypoxia; SkiErg; aerobic exercise; anaerobic exercise

## Abstract

**Background:**

The key factors for cross-country skier training are high-altitude acclimatization and strength in double-polling. This study aimed to identify the effects of artificially induced high altitude on acute performance changes during aerobic (AE) and anaerobic (ANE) threshold exercises. Sport-specific tests simulating double-poling cross-country skiing were performed.

**Methods:**

Eleven (8 ♂ and 3 ♀) highly trained cross-country skiers (age 19±2.81, BMI 21.9±2.1) performed a stress test to determine individual AE and ANE levels and AE and ANE intensity tests at low (500m ASL) and artificially induced high (2000m ASL) altitudes. The altitude was simulated using the hypoxic generator HYP-100™. For double-poling, the ergometer SkiErg ^®^ was used in the standing position. Heart rate (HR) and lactate concentration (LC) in the capillary blood were monitored.

**Results:**

For the AE and ANE loads, the average HR values at an artificially induced high altitude were 3–5% higher than those at a low altitude. The differences were significant, both statistically (p<0.05) and substantively. The average LC values were neither statistically significant (p>0.05) nor substantively significant.

**Conclusions:**

Not-adapted youth elite cross-country skiers have higher HR at artificially induced high altitudes than for the same load in lowlands. The absence of alterations in the average LC confirms that it is more appropriate to monitor the HR for altitude acute effect assessment and employ LC only for verification.

## Background

Currently, the methodology for preparing the best cross-country skiers focuses on the importance of strength training, not just for sprints ( Hébert-Losier et al., 2017) but also for long-distance tracks ( Sandbakk & Holmberg, 2014) and on the utilization of higher altitudes between 1 800 and 2 200 m above sea level ASL ( Suchý, 2012). For testing ( Fukuda et al., 2014; Hegge et al., 2015) and training of specific power endurance abilities in cross-country skiing, the SkiErg ^®^ Concept 2 device (Morrisville, VT, USA) was utilized. Since 2011, this simulator has been the standard for testing racers enrolled in the Youth Sports Centers of the Czech Ski Association. The rise of the strength-oriented concept of cross-country skiing is determined primarily by the sliding ability of skis, races taking place on artificial snow, and track design. It is not just World Cup (WC) races that are currently ceasing to be raced on natural snow, but many other races also take place on “firn” (almost ice) snow. Tracks are increasingly demanding to make races more attractive. Therefore, skiers with advanced strength have been more successful in recent years.

Double-poling cross-country skiing (hereinafter double-poling) has recently been discussed not only for long-distance races but also for easier (i.e., generally older) WC tracks. Under suitable conditions, some skiers could complete classic-technique long-distance WC races using double-poling (e.g., Dario Cologna and Petter Northug Jr. at Davos WC in the 2015/2016 season). The advantage of much more strength-intensive double-poling compared to classic cross-country skiing is the ability to complete the entire cross-country race without kick wax. Such skis are faster on downhill and flat sections, and even slightly inclined. In the 2016/2017 season, the Fédération Internationale de Ski (FIS) reacted to this rise in double-poling by shortening the pole length for classic cross-countries, with the rules now defining a length (max. 83% of the racers’ height in ski boots) is less favorable for double-poling. Furthermore, juries of certain races can now define what are called “classic zones” or “no double pole technique zones,” where double-poling is prohibited ( www.fis-ski.com). Nevertheless, double-poling has become a vital part of the preparation, with treadmill and double-poling ergometer being the most suitable training tools to measure this ability.

Several studies have been published on utilizing higher altitudes (1 800 2 400 m ASL) to increase physical endurance, along with competition results at low altitudes ( Millet & Schmitt, 2011; Suchý & Opočenský, 2015; Wilber, 2004). A meta-analysis by Bonetti and Hopkins (2009) confirmed that after a three-week sojourn at natural altitude, top athletes, on average, increased their performance upon returning to low altitudes by 5.2%. Higher altitudes place greater demands on training management than on training at sea level, especially for adult elite athletes ( Burtscher et al., 2018). Therefore, redefining the exercise intensities obtained at low altitudes before the onset of acclimatization processes for the first days of stay and altitude training is essential. Heart rate (HR) and lactate concentration (LC) in capillary blood may be significantly higher at the same exercise intensity as at sea level, which is generally called the “lactate paradox” ( Hochachka et al., 2002; Lundby et al., 2000). Long training camps at altitude, essentially for the whole preparatory period and even part of the competition period, are necessary for top skiers ( Chrástková & Suchý, 2011). Subsequently, due to the frequent absence of snow at low altitudes ( Dong & Menzel, 2020), cross-country skier training at the start of the season is increasingly shifting to higher altitudes of 1 800 to 2 200 m ASL. This has negative socio-economic consequences. Thus, to a limited extent, alternatives have been used at artificially induced altitudes. Several studies have described its positive effects ( Treff et al., 2022; Pupiš et al., 2014; Wilber, 2004). Artificial higher altitudes used for improving physiological parameters can be induced by masks, oxygen tents, or “Alpine houses.”

For sports training, a simplified distinction of three primary exercise intensities is used: aerobic, anaerobic, and critical ( Bunc, 1990; Keul et al., 1978). With regard to the importance of indicating changes in acute performance at various altitudes and exercise intensities before the onset of acclimatization processes, we focused on this issue in our study. For verification, we utilized the SkiErg machine because only a few studies have been conducted, despite its wide application in the field and beyond. We identified only a single study ( Zinner et al., 2015) that dealt with performance changes in artificially induced altitude using double-poling ergometer.

This study aimed to determine the impact of artificially induced altitude (2,000 m ASL) on acute performance changes in aerobic and anaerobic threshold levels. For testing, imitation of double-poling was used on elite cross-country skiers via a sport-specific test on the ski double-poling ergometer. We focused on the lactate concentration (LC) and heart rate (HR) values for aerobic and anaerobic intensities. We hypothesized that the differences in these parameters at both altitudes would be statistically significant and have a medium effect size.

## Methodology

### Participants

The study was conducted on a homogeneous group of highly trained young cross-country skiers, n = 11 (8 ♂ and 3 ♀). The characteristics of the subjects are shown in Table 1. All participants regularly (at least four annual training cycles, ATC) attended the races of the Czech Cross-Country Cup (cca 8 distance and four sprint races during ATC). Most of them were part of the national team. All the participants were in the first part of the preparatory period during the experiment. They underwent training exercises of 15–20 hours per week. During testing, all the subjects were healthy and free to step out of the experiment at any time.

**Table 1.** Characteristics of subjects.

	Fat [%]	Weight [kg]	Height [cm]	BMI	Age [years]	Lung capacity [l]	FEV1	Training per ATC [hrs]
** Mean ± SD**	11.8 ** ± **2.76	68.5 ± 9.22	181 ± 0.08	21.9 ± 2.1	19 ± 2.81	5.35 ± 1.15	4.4 ± 0.75	581 ± 123

BMI - Body mass index, FEV1- Forced expiratory volume in 1 second, ATC - Annual training cycle.

### Intervention

**Figure 1.**
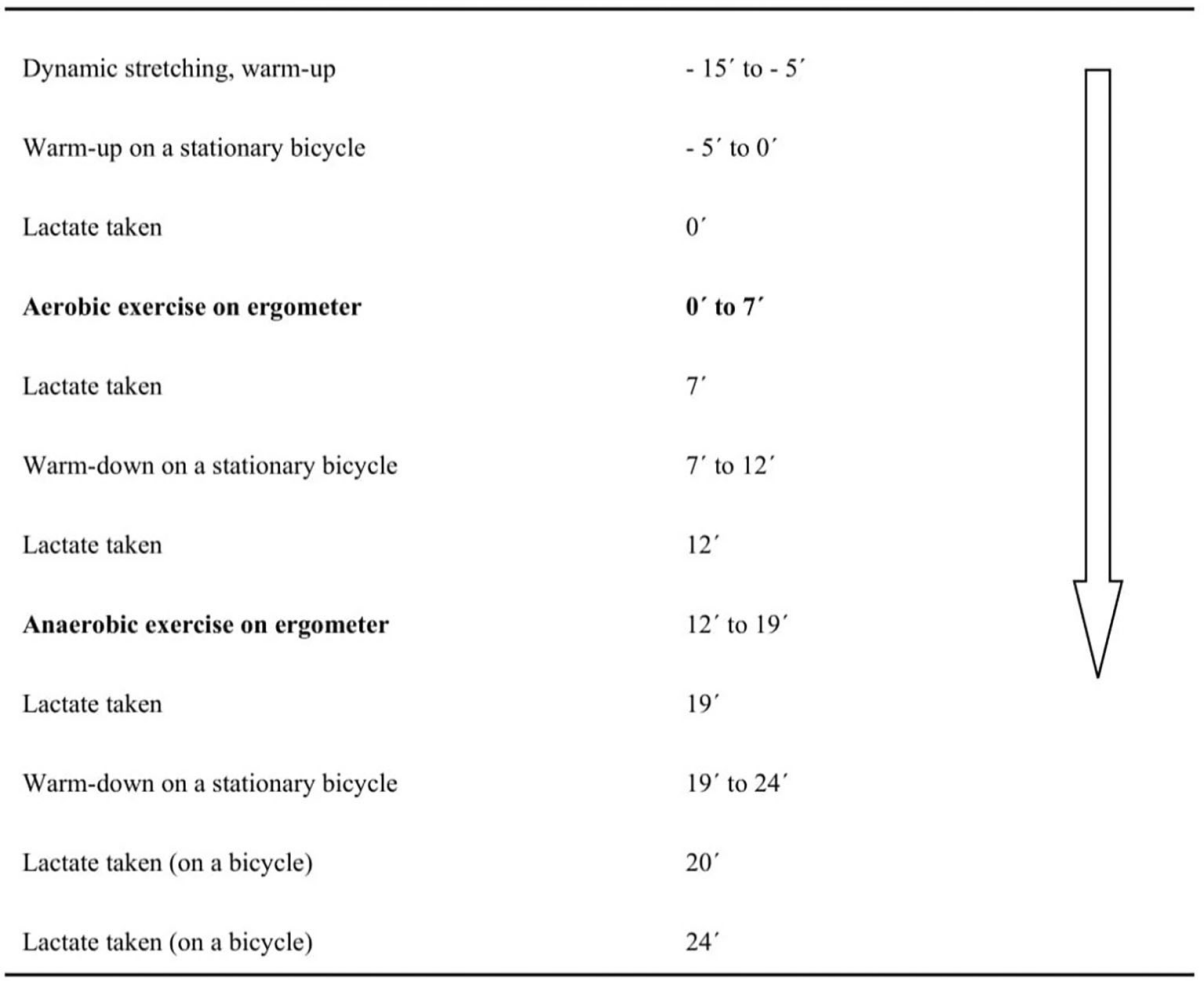
Timeline of testing on double-poling ergometer.

A timeline of the experiment is shown in Figure 1. In total, three imitated double-poling tests were performed using the double polling ergometer machine and a hypoxic generator over three consecutive days:

1. The graded stress test was modified according to Buskirk et al. (1967) and Faulkner et al. (1968) to determine individual AE and ANE values.
2. Non-standardized aerobic and anaerobic intensity test at low altitude (500 m ASL).
3. Non-standardized aerobic and anaerobic-intensity test at artificially induced high altitude (2 000 m ASL).

The individual resistance during the first test was defined based on a graded stress test. During all tests, the subjects upheld the defined intensity of exercise using an electronic display with information on power in watts and heart rate (HR).

All subjects underwent all three tests at the preset intensity. The temperature of the room during testing was 22 – 24 °C. During all the tests, the subjects breathed exclusively through a mask with a supply of air from a hypoxic generator. Before testing, all participants were informed in detail about the technique of using the ergometer, the necessity of constantly checking that the mask was tight, and the necessity of completing the tests responsibly and with the required effort. The subjects regularly used double-poling ergometer during their training. Following recommendations ( Pacholek 2021, 2023; Pacholek & Zemková, 2022), the research team members actively motivated all subjects verbally to the same level throughout the tests.

In accordance with the recommendations of SkiErg’s manufacturer, the technique for imitating double poling during cross-country skiing is as follows:

1. Standing with hands and feet shoulder-width apart and hands slightly above the head, arms bent at the elbows.
2. The subjects drove the handles downward by engaging their core abdominal muscles and bending their knees; they maintained the bend in their arms to keep the handles fairly close to their face.
3. They finished the drive with knees moderately bent and arms extended alongside their thighs.
4. They extended their arms upward and straightened their bodies to return them to the starting position.
5. If technical violations were observed, they were immediately corrected verbally by invigilators.

The machine’s resistance is directly proportional to the force exerted by the athlete; the greater the force expended, the greater the resistance created by the ergometer (measured in watts). This double-poling ergometer ( www.concept2.com) utilizes the resistance of air set by a valve, but the amount of air flowing into the gyroscope does not impact the resistance, only the cadence of the pulls. The valve was set at level 7 for the entire period for all subjects. Performance was continually recorded in the ergometer’s memory and portable USB drive, making it possible to analyze the exercise.

The hypoxic generator HYP-100™ (Hypoxico Inc., USA) was used to induce artificial hypoxia. Before testing, the hypoxic generator was tested using a Servomex 1440 device (Servomex Group Ltd., Crowborough, UK) at the Biomedical Lab (Faculty of Physical Education and Sports, Charles University), and the data were read according to the manufacturer’s instructions. An exercise bike, Hause Fit (Ontario, CA, USA), was used for cooling and regeneration. The Polar S810 device (Polar Electro Oy, Kempele, Finland) continuously monitored the HR. LC was analyzed from capillary blood samples obtained from the finger using a SensoStar GL device (DiaSys, Holzheim, Germany). According to the manufacturer, it was measured with a variation coefficient of <2.5% for 24 samples (each with a volume of 90 mg.dl ^−1^). Vital lung capacity was assessed using a MIR Spirobank II analyzer (MIR Inc. Roma, Italy). The manufacturer claimed an accuracy of ±3%, or 50 ml. Anthropometry was performed by an experienced person using the skinfold measurement method. Relative body fat percentage can be estimated by measuring skinfold thickness at five sites: biceps, triceps, subscapular, suprailiac, and medial calf. The measurements are summed, multiplied by two, and the result is compared to age- and sex-specific reference tables to determine body fat percentage. This method is practical, non-invasive, and reliable when performed using standardized techniques ( Parizkova 1961).

### Statistical analysis

The statistical significance of the mean differences of monitored values was determined using a parametric paired t-test for two dependent samples at the level of significance (p < 0.05) ( Jaroš et al., 1998). Substantive significance was evaluated by Cohen’s d, then assessing the size of the effect according to Hendl (2009): over 0.8, large effect; 0.5 – 0.8, medium effect; 0.2 – 0.5, small effect; and less than 0.2, negligible effect. The normality of the dataset distribution was analyzed using the Kolmogorov–Smirnov and Shapiro–Wilk tests.

## Results

Individual LC curves, including AE and ANE intensity, were established based on the initial graded stress test on double-poling ergometer. The determined intensities were then verified expertly by the racers’ trainers, who adjusted the intensity for two participants by 5% and 7%. These individuals underwent exercise at defined aerobic and anaerobic intensities at low (500 m ASL) and artificially induced high altitudes of 2 050 m ASL afterward.

Table 2 reports the mean lactate values during aerobic and anaerobic intensity exercise. Table 3 reports the mean HR values in AE, and Table 4 reports the mean heart rate values in ANE.

**Table 2.** Lactate values.

	At rest	Immed. after AE	5 min after AE	Immed. after ANE	1 min. after ANE	5 min. after ANE
Mean ± SD 500 m ASL [mmol.l ^−1^]	1.27 ± 0.4	2.59 ± 0.6	1.43 ± 0.4	5.05 ± 1.2	4.44 ± 0.8	3.19 ± 0.5
Mean ± SD 2 000 m ASL [mmol.l ^−1^]	1.08 ± 0.3	2.57 ± 0.8	1.41 ± 1.2	5.29 ± 1.4	4.65 ± 1.2	3.36 ± 1.0
% diff. 500/2 000 m ASL	-16.5%	-0.8%	-1.7%	4.4%	4.9%	5.2%
t-test 500/2 000 m ASL	0.05	0.47	0.44	0.31	0.25	0.31
d – substantive signif.	0.2	0.0	0.0	0.1	0.1	0.1

AE - aerobic intensity exercise, ANE - anaerobic intensity exercise, SD - standard deviation, ASL - above sea level, mmol.l ^−1^ - millimoles per liter.

**Table 3.** Heart rate values in aerobic intensity.

	HR 2-3 min of AE	HR 4-5 min of AE	HR 6-7 min of AE	HR 1 min. after AE	HR 2 min. after AE
Mean ± SD 500 m ASL	115.5 ± 11.7	116.1 ± 11.8	117.5 ± 11.4	96.4 ± 6.4	92.1 ± 8.5
Mean ± SD 2 000 m ASL	119.3 ± 13.2	121.5 ± 13.7	123.7 ± 14.3	98.6 ± 10.2	93.1 ± 9.6
% diff. 500/2 000 m ASL	3.3%	4.6%	5.3%	2.4%	1.1%
t-test 500/2 000 m ASL	0.06	0.02*	0.01*	0.20	0.27
d – substantive signif.	0.3	0.5	0.6	0.0	0.0

HR - heart rate in one minute, AE - aerobic intensity exercise, SD - standard deviation, ASL - above sea level.

**Table 4.** Heart rate values in anaerobic intensity.

	HR before the start of ANP	HR 2-3 min of ANP	HR 4-5 min of ANP	HR 6-7 min of ANP	HR 1 min. after ANP	HR 2 min. after ANP
Mean ± SD 500 m ASL	94.4 ± 10.6	144.7 ± 16.1	148.1 ± 15.4	151.5 ± 15.5	113.9 ± 12.1	105.4 ± 11.5
Mean ± SD 2 000 m ASL	90.0 ± 10.1	148.8 ± 16.8	153.7 ± 16.1	157.7 ± 15.5	115.6 ± 14.1	106.1 ± 12.8
% diff. 500/2 000 m ASL	-4.7%	2.8%	3.6%	4.1%	1.4%	0.7%
t-test 500/2 000 m ASL	0.26	0.05*	0.02*	0.00*	0.27	0.31
d – substantive signif.	0.2	0.8	0.9	0.8	0.2	0.1

HR - heart rate in one minute, ANE - anaerobic intensity exercise, SD - standard deviation, ASL - above sea level.

The differences in the average LC during and following the tests at aerobic and anaerobic intensities ( Table 2) at both altitudes were neither statistically significant (p > 0.05) nor substantively significant. We did not evaluate the LC level after warming up, as it is not sufficiently reliable, although we attempted to manage the course of warming up as a standard.

The average HR values of all tests at high altitudes were consistently statistically significant compared to those at low altitudes (p < 0.05). The substantive significance of the differences was at the boundary of the medium-to-large effect.

The calming kinetics in the first and second minutes after AE and ANE were not statistically significant (p < 0.05) or substantively significant.

## Discussion

Our study showed that the high altitude HR values were statistically (p < 0.05) and substantively higher in lowland areas at both the AE and ANE levels. For the average lactate values, no statistically significant or substantively significant differences were reported. The data partially correspond to our prior studies ( Suchý, 2012), with the one difference being that the acclimatization of the athletes preceded the tests conducted at high altitudes. The “lactate paradox” ( Hochachka et al., 2002; Lundby et al., 2000) was not discovered in our study. This could be because our participants did not undergo maximum-intensity exercise.

The results demonstrate that an acute artificially induced altitude of 2000 m ASL among the monitored cross-country skiers led to an increased internal load intensity indicated by HR. The general findings of this test correspond to those of previously published studies. However, these methods do not utilize artificially induced altitudes or a double-poling ergometer. It has been confirmed that adapted individuals have a 3–5% higher HR at a higher altitude during the same load as at sea level ( Fuchs & Reiss, 1990; Powel & Garcia, 2000; Pupiš et al., 2014; Truijens et al., 2008; Wilber, 2004). An interesting indicator at altitude without prior adaptation is the average HR value between minutes 6 and 7 for ANP, where the value at the artificially induced altitude of 2,000 m ASL was six beats higher than at sea level. This value confirms the necessity of realizing adaptation processes before commencing training exercises of the same intensity at low altitudes ( Millet & Schmitt, 2011; Wilber, 2004). Furthermore, the results confirm that HR is more suitable than LC for monitoring the immediate reaction of a body that is not adapted to higher altitudes in capillary blood. This fact is consistent with publications reporting an increase in HR during maximum load by 10% to 30% in individuals not adapted to altitude ( Bonetti & Hopkins, 2009; Millet & Schmitt, 2011; Suchý, 2012; Wilber, 2004).

These results correspond to those reported by Zinner et al. (2015). The study reported changes in performance during artificially induced high altitudes and lowlands using double-poling ergometer. The test design lasted for 3 × 3 min. of maximum intensity exercise, with the subjects also having hypoxic conditions during the break, in contrast to our design. Sandbakk et al. (2015) performed all-out tests on a double-poling ergometer machine in highly trained athletes who were sitting. This testing method differs fundamentally from ours; thus, no comparison is possible. Testing subjects in standing positions is more appropriate for a better simulation of cross-country skiing. Fukuda et al. (2014) used double-poling ergometer to confirm a multicriteria accord of results in 3 × 3 min all-out tests with a maximum load of 300 m, 650 m, and 1 000 m. Therefore, we considered the design of our study appropriate.

The 7-minute AE and ANE performance test segments corresponded to the subjects’ current fitness level. The altitude of 2000 m ASL was selected for its contribution to practical application, and the possibility that results in the tests could have improved with optimization of the technique of pulling, thus degrading the results, can be ruled out because all participants regularly used the ergometer for training and tests. The double-poling ergometer tests are also part of the test battery of the Czech Ski Association.

We did not analyze the intersexual differences in the results because the study was conducted on a small set and the goal was to compare the differences in exercise at low and high altitudes. However, no significant differences in the performance of men and women during the all-out tests on double-poling ergometer were identified by Hegge et al. (2015).

In the structured post-test interviews, the participants did not report any problems with the masks. They also reported no complications related to inhaling relatively dry air from the hypoxic generator. The reason is their experience with this type of mask and the dry air from the functional examinations they undergo several times per annual training cycle.

This study has some limitations. It was impossible to test the impact of acute changes in altitude on the critical intensity. Unfortunately, this intensity could not be measured because of the limited capacity of the hypoxic generator. For this assessment, it is necessary to use (at least) two hypoxic generators and acquire larger sacs that are not supplied by the manufacturer. Information on critical intensity exercise at altitude could have interesting benefits for application, for example, as an input for training management during unplanned shifts to altitude or arriving at races at altitude without prior adaptation. We consider the use of face masks as a limiting factor in the study of equipment. Although we used a very high-quality rubber band, we were forced to attach it to a second rubber band so that it fit snugly. Despite all efforts to affix the mask to the face perfectly, there is a certain risk that for female subjects, the test could have been slightly influenced by the mask not being tight, thus inhaling partially normoxic ambient air. We minimized this risk by performing regular checks. The experts set the initial load values for the graded stress tests. The initial value was set improperly for two subjects, so it was necessary to repeat this test. No statistically or substantively significant change was found immediately before the second test, but in light of the fact that the value being investigated just before the test, and the subjects had been 5 min without the mask providing air from the hypoxic generator, we do not consider this value essential. The AE and ANE were defined based on a sea-level graded stress test. If we were to choose a precise term for the exercise at an artificially induced altitude, then the exercise the athletes completed here should not be called such. For a better understanding, we can use oxygen saturation measurements. Another limitation is the methodology determination of AE and ANE by Buskirk et al. (1967) and Faulkner et al. (1968), which has been questioned in the current literature, for example, by Poole et al. (2021). The “classical” methodology that we applied is frequently used as a part of the training process management in cross country skiing.

Based on our investigation and past studies, we are considering a similar study design at natural altitudes, that is, without the use of hypoxic generators. In this way, the study could be expanded to include the speed of acclimatization processes in confrontation with the drop in strength endurance and purely endurance performance at higher altitudes compared to testing at sea level.

## Conclusion

Not-adapted elite young cross-country skiers have higher HR at artificially induced high altitudes than for the same load in lowlands. The absence of alterations in the average LC confirms that it is more appropriate to monitor the HR for altitude acute effect assessment and employ LC only for verification.

### Bioethical clearance

The study was designed in accordance with the Helsinki Declaration and approved by the Ethics Committee of Charles University, Faculty of Physical Education and Sport (reference number 115/2015). Before the study was launched, all subjects were acquainted with the course in detail and subsequently signed an informed consent form. The authors declare no conflicts of interest related to this study.

## Data Availability

The data presented in this study are available upon request from the corresponding author (email - pbartik@psu.edu.sa). The data were not publicly available because of ethical committee restrictions and the preservation of participant privacy.

## References

[ref1] Bonetti DL Hopkins WG : Meta-analysis of sea level performance following adaptation to hypoxia. *Sports Med.* 2009;39:107–127. 10.2165/00007256-200939020-00002 19203133

[ref2] Bunc V : *Biokybernetický přístup k hodnocení reakce organismu na tělesné zatížení.* Praha: Univerzita Karlova;1990.

[ref3] Burtscher M : Preparation for Endurance Competitions at Altitude: Physiological, Psychological, Dietary and Coaching Aspects. A Narrative Review. *Front. Physiol.* 2018;9:1504. 10.3389/fphys.2018.01504 30425646 PMC6218926

[ref4] Buskirk ER : Maximal performance at altitude and on return from altitude in conditioned runners. *J. Appl. Physiol.* 1967;23:259–266. 10.1152/jappl.1967.23.2.259 6033527

[ref13] Chrástková M Suchý J : Názory trenérů lyžařů běžců na přípravu ve vyšší nadmořské výšce. *Studia Sportiva.* 2011;1:145–153.

[ref5] Dong C Menzel L : Recent snow cover changes over central European low mountain ranges. *Hydrol. Process.* 2020;34(2):321–338. 10.1002/hyp.13586

[ref6] Faulkner B : Maximum aerobic capacity and running performance at altitude. *J. Appl. Physiol.* 1968;24:685–691. 10.1152/jappl.1968.24.5.685 5647647

[ref7] Fuchs U Reiss M : *Höhentraining: das erfolgs konzept der ausdauer sportarten (Trainerbibliothek 27).* Münster: Philippkaverlag;1990.

[ref8] Fukuda DH : Characterization of the work–time relationship during cross-country ski ergometry. *Physiol. Meas.* 2014;35:31–43. 10.1088/0967-3334/35/1/31 24345800

[ref9] Hébert-Losier K : Factors that Influence the Performance of Elite Sprint Cross-Country Skiers (Review). *Sports Med.* 2017;47(2):319–342. 10.1007/s40279-016-0573-2 27334280 PMC5266777

[ref10] Hegge AM : Gender differences in power production, energetic capacity and efficiency of elite cross-country skiers during whole-body, upper-body, and arm poling. *Eur. J. Appl. Physiol.* 2015;116:291–300. 10.1007/s00421-015-3281-y 26476546

[ref11] Hendl J : *Přehled statistických metod: analýza a metaanalýza dat.* Praha: Portál; 3., přeprac. Vyd 2009.

[ref12] Hochachka PW : The lactate paradox in human high-altitude physiological performance. *News Physiol. Sci.* 2002;17:122–126. 12021383 10.1152/nips.01382.2001

[ref14] Jaroš F : *Pravděpodobnost a statistika (skriptum).* Praha: VŠCHT;1998.

[ref15] Keul J : Die aerobe und anaerobe Kapazität grundlage für die leistungs Diagnostik. *Leistungssport.* 1978;1:22–32.

[ref16] Lundby C Saltin B Van Hall G : The “lactate paradox” evidence for a change in the course of acclimatization to severe hypoxia in lowlanders. *Acta Physiol. Scand.* 2000;179:265–269. 10.1046/j.1365-201x.2000.00785.x 11450136

[ref17] Millet G Schmitt L : *S‘entraîner en altitude, Mécanismes, méthodes, exemples, conseils pratiques.* Brussel: De Boeck;2011.

[ref20] Pacholek M : The effects of various stimuli on motivation and physical fitness of physically active and non-active students. *Ann. Appl. Sport Sci. * 2021;9(4). 10.52547/aassjournal.954

[ref18] Pacholek M : The influence of verbal encouragement on heart rate, maximum oxygen uptake, and distance covered in young male adults during beep test. *J. Mens Health.* 2023;19(2):29–35.

[ref19] Pacholek M Zemková E : Effects of verbal encouragement and performance feedback on physical fitness in young adults. *Sustainability.* 2022;14(3):1753. 10.3390/su14031753

[ref21] Parizkova J : Total body fat and skinfold thickness in children. 1961. 14483890

[ref22] Poole DC : The anaerobic threshold: 50+ years of controversy. *J. Physiol.* 2021;599(3):737–767. 10.1113/jp279963 33112439

[ref23] Powel FL Garcia N : Physiological effects on intermittent hypoxia. *Altitude Medicine and Biology.* 2000;1:125–136. 10.1089/15270290050074279 11256564

[ref24] Pupiš M : *World research of hypoxic training.* Bánská Bystrica: UMB;2014.

[ref25] Sandbakk Ø Holmberg HC : A reappraisal of success factors for Olympic cross-country skiing. *Int. J. Sports Physiol. Perform.* 2014;9(1):117–121. 10.1123/ijspp.2013-0373 24088346

[ref32] Sandbakk Ø Skålvik TF Spencer M : The physiological responses to repeated upper-body sprint exercise in highly trained athletes. *Eur. J. Appl. Physiol.* 2015;115(6):1381–1391. 25677383 10.1007/s00421-015-3128-6

[ref26] Suchý J : *Využití hypoxie a hyperoxie ve sportovním tréninku.* Praha: Karolinum;2012.

[ref27] Suchý J Opočenský J : Usefulness of training camps at high altitude for well-trained adolescents. *Acta Universitatis Palackianae Olomucensis Gymnica.* 2015;45:13–20. 10.5507/ag.2015.002

[ref28] Treff G Sareban M Schmidt WFJ : Hypoxic Training in Natural and Artificial Altitude. *German Journal of Sports Medicine/Deutsche Zeitschrift fur Sportmedizin.* 2022;73(3):112–117. 10.5960/dzsm.2022.529

[ref29] Truijens MJ : The effect of intermittent hypobaric hypoxic exposure and sea level training on submaximal economy in well-trained swimmers and runners. *J. Appl. Physiol.* 2008;104:328–337. 10.1152/japplphysiol.01324.2006 18048583

[ref30] Wilber RL : *Altitude training and Athletic Performance.* Champaign: Human Kinetics;2004.

[ref31] Zinner C : Influence of Hypoxic Interval Training and Hyperoxic Recovery on Muscle Activation and Oxygenation in Connection with Double- Poling Exercise. *PloS ONE.* 2015;10(10):e0140616. 10.1371/journal.pone.0140616 26468885 PMC4607305

