# Supplementary material for: Artificially induced altitudes aerobic and anaerobic performance differences on double-poling ergometer in elite young cross-country skiers
Source: F1000Res. 2025 Oct 6;14:744. Originally published 2025 Jul 28. [Version 2] doi: 10.12688/f1000research.166884.2 (PMC12648023; doi:10.12688/f1000research.166884.2)
Supplement: Peer review history

# Reviewer report: Reviewer response for version 2

**DOI:** 10.5256/f1000research.188535.r427635

**Published:** 2025-11-25

**Authors:** Juris Grants

**Affiliations:** 1 Riga Stradins University, Latvian Academy of Sport Education, Riga, Latvia

 Cross-country skiing competitions in high altitudes are now a well-known fact, but at the same time, require more attention for deeper research. Practical research, skillfully selected research methods, the study is structured step by step, sequentially, to arrive at specific conclusions, positively developed recommendations for trainers, and adherence to the principles of acclimatization.

Is the work clearly and accurately presented and does it cite the current literature?

Yes

If applicable, is the statistical analysis and its interpretation appropriate?

I cannot comment. A qualified statistician is required.

Are all the source data underlying the results available to ensure full reproducibility?

No source data required

Is the study design appropriate and is the work technically sound?

Yes

Are the conclusions drawn adequately supported by the results?

Yes

Are sufficient details of methods and analysis provided to allow replication by others?

Yes

Reviewer Expertise:

Health and Sport Science, sport didactics

I confirm that I have read this submission and believe that I have an appropriate level of expertise to confirm that it is of an acceptable scientific standard.

# Reviewer report: Reviewer response for version 1

**DOI:** 10.5256/f1000research.183936.r404694

**Published:** 2025-09-10

**Authors:** David Liska

**Affiliations:** 1 Matej Bel University, Banská Bystrica, Slovakia

The manuscript addresses a relevant and specific topic in sports science, acute physiological responses to artificially induced high altitude in elite young cross-country skiers, measured via a sport-specific test on double-poling ergometer (Skierg).

 1.More background information, more information in methods and results section is needed in abstract     

 2. Is very funny opinion “Currently, the methodology for preparing the best cross-country skiers focuses on the importance of strength training, not just for sprints ( Hébert-Losier et al., 2017) Ref 1 but also for long-distance tracks”, did coach from cross country skier check manuscript?

 3. Is 2000m is real hypoxic attitude?

 4. Why you write about double-polling techniques when you write article about attitude stimulation?

 “Double-poling cross-country skiing (hereinafter double-poling) has recently been discussed not only for long-distance races but also for easier (i.e., generally older) WC tracks. Under suitable conditions, some skiers could complete classic-technique long-distance WC races using double-poling (e.g., Dario Cologna and Petter Northug Jr. at Davos WC in the 2015/2016 season). The advantage of much more strength-intensive double-poling compared to classic cross-country skiing is the ability to complete the entire cross-country race without kick wax. Such skis are faster on downhill and flat sections, and even slightly inclined. In the 2016/2017 season, the Fédération Internationale de Ski (FIS) reacted to this rise in double-poling by shortening the pole length for classic cross-countries, with the rules now defining a length (max. 83% of the racers’ height in ski boots) is less favorable for double-poling. Furthermore, juries of certain races can now define what are called “classic zones” or “no double pole technique zones,” where double-poling is prohibited ( www.fis-ski.com). Nevertheless, double-poling has become a vital part of the preparation, with treadmill and double-poling ergometer being the most suitable training tools to measure this ability.”

 5. Why is lactate concertation is important? “Heart rate (HR) and lactate concentration (LC) in capillary blood may be significantly higher at the same exercise intensity as at sea level, which is generally called the “lactate paradox””

 6. Are you sure? “For sports training, a simplified distinction of three primary exercise intensities is used: aerobic, anaerobic, and critical ( Bunc, 1990; Keul et al., 1978). “, it is possible get anaerobic conditions? Is not myth?

 7. the same situation, Is anaerobic threshold real condition? “anaerobic threshold levels. “

 8. more precision sport loading is needed “All the participants were in the first part of the preparatory period during the experiment. They underwent training exercises of 15–20 hours per week.”

 9. Table 1 is so short

 10. Are you sure ? 1967 and 1968…

 11. The graded stress test was modified according to Buskirk et al. (1967) and Faulkner et al. (1968) to determine individual AE and ANE values.

 12. Why is lactate important? Table 2

 13. Check by native speaker

Is the work clearly and accurately presented and does it cite the current literature?

Partly

If applicable, is the statistical analysis and its interpretation appropriate?

Partly

Are all the source data underlying the results available to ensure full reproducibility?

Yes

Is the study design appropriate and is the work technically sound?

Partly

Are the conclusions drawn adequately supported by the results?

Yes

Are sufficient details of methods and analysis provided to allow replication by others?

Partly

Reviewer Expertise:

sport science

I confirm that I have read this submission and believe that I have an appropriate level of expertise to confirm that it is of an acceptable scientific standard, however I have significant reservations, as outlined above.

## References

[1] : Updated reliability and normative values for the standing heel-rise test in healthy adults. *Physiotherapy* .2017;103(4) : 10.1016/j.physio.2017.03.002 446-452 10.1016/j.physio.2017.03.002 28886865

# Response

**Published:** 2025-09-20

**Authors:** Dr. Peter Bartik

1.More background information, more information in methods and results section is needed in abstract    

**We added more information in background and results section. We think, that methods are sufficiently described.**

 2. Is very funny opinion “Currently, the methodology for preparing the best cross-country skiers focuses on the importance of strength training, not just for sprints (Hébert-Losier et al., 2017) Ref 1 but also for long-distance tracks”, did coach from cross country skier check manuscript?

**We agree: we have edited the sentence**

 3. Is 2000m is real hypoxic attitude?

**Yes: 2,000 m ASL is considered the hypoxic altitude (f. e. Burtscher et al., 2018)**

 4. Why you write about double-polling techniques when you write article about attitude stimulation?

  “Double-poling cross-country skiing (hereinafter double-poling) has recently been discussed not only for long-distance races but also for easier (i.e., generally older) WC tracks. Under suitable conditions, some skiers could complete classic-technique long-distance WC races using double-poling (e.g., Dario Cologna and Petter Northug Jr. at Davos WC in the 2015/2016 season). The advantage of much more strength-intensive double-poling compared to classic cross-country skiing is the ability to complete the entire cross-country race without kick wax. Such skis are faster on downhill and flat sections, and even slightly inclined. In the 2016/2017 season, the Fédération Internationale de Ski (FIS) reacted to this rise in double-poling by shortening the pole length for classic cross-countries, with the rules now defining a length (max. 83% of the racers’ height in ski boots) is less favorable for double-poling. Furthermore, juries of certain races can now define what are called “classic zones” or “no double pole technique zones,” where double-poling is prohibited (www.fis-ski.com). Nevertheless, double-poling has become a vital part of the preparation, with treadmill and double-poling ergometer being the most suitable training tools to measure this ability.”

 Using of SkiErg in the training process of skiers is primarily focused on improving performance during double poling. Elite cross country systematically use high altitude in preparation

 5. Why is lactate concertation is important? “Heart rate (HR) and lactate concentration (LC) in capillary blood may be significantly higher at the same exercise intensity as at sea level, which is generally called the “lactate paradox””

 Lactate concentration is (together with heart rate) the most commonly used parameter for monitoring and analyzing training load in endurance sport – we include this information in the article

 6. Are you sure? “For sports training, a simplified distinction of three primary exercise intensities is used: aerobic, anaerobic, and critical (Bunc, 1990; Keul et al., 1978). “, it is possible get anaerobic conditions? Is not myth?

**We included in the chapter discussion paragraph about terminology (included citation Soll et al., 2017)**

 7. the same situation, Is anaerobic threshold real condition? “anaerobic threshold levels. “

**We included in the chapter discussion paragraph about terminology (included citation Soll et al., 2017)**

 8. more precision sport loading is needed “All the participants were in the first part of the preparatory period during the experiment. They underwent training exercises of 15–20 hours per week.”

**We included detailed information about proband training structure during testing period: roller ski: 30%, running: 50%, cycling: 10%, strength: 10%, 90% AP and 10% ANP training intensity**

 9. Table 1 is so short

**Yes: contains only key information**

 10. Are you sure ? 1967 and 1968…

**We included information, that we used standard test using by Ski Association of the Czech Republic (Cross country section), which was modified based on Buskirk et al. (1967) , Faulkner et al. (1968) and Poole et al. (2021)**

 11. The graded stress test was modified according to Buskirk et al. (1967) and Faulkner et al. (1968) to determine individual AE and ANE values.

**We included information, that we used standard test using by Ski Association of the Czech Republic (Cross country section),**

 12. Why is lactate important? Table 2

**Lactate analysis is (together with heart rate) the most commonly used parameter for monitoring and analyzing training load in endurance sport**

# Reviewer report: Reviewer response for version 1

**DOI:** 10.5256/f1000research.183936.r402544

**Published:** 2025-09-02

**Authors:** Vassilios Panoutsakopoulos

**Affiliations:** 1 Aristotle University of Thessaloniki, Thessaloniki, Greece

In the submitted manuscript, the authors studied the effects of artificially induced high altitude on acute performance changes during aerobic (AE) and anaerobic (ANE) threshold exercises in elite young cross-country skiers with a use of a double-poling cross-country skiing simulator. Results indicated that average HR values at an artificially induced high altitude were higher compared to the lower altitude, but no differences were observed for lactate concentration.

 The topic is of interest but there are issues that need to be addressed, as mentioned in the following comments.

**Comments **

1. Abstract = Background: it is recommended to use “Among the key factors…”; Results for HR: please, clarify “both statistically (p<0.05) and substantively” (mainly the latter).
2. Background = Further elaboration is recommended regarding the examined parameters (HR, La, body fat) in the context of work physiology and their impact in cross-country skiing performance. In addition, further information is required for the introduction of the aerobic and anaerobic threshold levels. This will also help to strengthen the rationale of the study. Finally, provide details why body fat will be assessed in this study and its effect on both actual cross-country skiing performance and measures.
3. Methodology = Provide the rationale to set the valve at level 7 for all participants rather than to provide an individualized resistance. In addition, further information is required for the skinfold measures (caliper details, equation/method used to estimate body fat). Also, provide further information about how the AE and ANE were set. Finally, note these specific comments: “the greater the force applied, the greater the resistance created by the ergometer” and “The measurements were summed”.
4. Statistical analysis = Provide evidence to establish the use of parametric tests, namely which one of the two tests was used. In addition, include information if a statistical software was used.
5. Results = Please, clarify “substantively”. Also, depict the results for bodyfat.
6. Discussion = cite past research findings related to the outcome of this study and the mechanisms involved, especially in 6 ^th^ and 7 ^th^ paragraph. In addition, add in the paragraph with the limitations of the study the small sample size and the lack to establish intersexual differences. Finally, add a final paragraph with the strengths of the study and the recommendations for future research.
7. Conclusion = add a couple of practical implications for coaches and practitioners.

Is the work clearly and accurately presented and does it cite the current literature?

Partly

If applicable, is the statistical analysis and its interpretation appropriate?

Partly

Are all the source data underlying the results available to ensure full reproducibility?

Yes

Is the study design appropriate and is the work technically sound?

Yes

Are the conclusions drawn adequately supported by the results?

Partly

Are sufficient details of methods and analysis provided to allow replication by others?

Partly

Reviewer Expertise:

Sports Biomechanics

I confirm that I have read this submission and believe that I have an appropriate level of expertise to confirm that it is of an acceptable scientific standard, however I have significant reservations, as outlined above.

# Response

**Published:** 2025-09-20

**Authors:** Dr. Peter Bartik

Comments

 1.Abstract = Background: it is recommended to use “Among the key factors…”;

**It was adjusted**

 2.Results for HR: please, clarify “both statistically (p<0.05) and substantively” (mainly the latter).

**It is clarified in statistical analysis section**

 3.Background = Further elaboration is recommended regarding the examined parameters (HR, La, body fat) in the context of work physiology and their impact in cross-country skiing performance. In addition, further information is required for the introduction of the aerobic and anaerobic threshold levels. This will also help to strengthen the rationale of the study. Finally, provide details why body fat will be assessed in this study and its effect on both actual cross-country skiing performance and measures.

**Information were included in chapter background (including citations)**

**Accurate identification of load intensity is crucial for proper training management (not only) in cross-country skiing, because load manipulation cannot be based on mere estimation (Neumann et al, 1998). Various stress tests are used to continuously verify fitness levels and determine the actual intensity at which the load was completed, which are evaluated using HR or La, for example (Bakhareva, 2022, Wilmore et al., 2008).**

**BMI values are currently widely discussed in cross-country skiing (Zopirrolli et al., 2020), as optimal weight has a significant impact on performance in tests and competitions.**

 4.Methodology = Provide the rationale to set the valve at level 7 for all participants rather than to provide an individualized resistance. In addition, further information is required for the skinfold measures (caliper details, equation/method used to estimate body fat). Also, provide further information about how the AE and ANE were set. Finally, note these specific comments: “the greater the force applied, the greater the resistance created by the ergometer” and “The measurements were summed”.

**We delete wrong comments: “the greater the force applied, the greater the resistance created by the ergometer”**

**We included information that level 7 probands typically use for training.**

**The skinfold measures were according to the methodology (Pařízková, 1961)**

**We included information how the AE and ANE were setting**

 5.Statistical analysis = Provide evidence to establish the use of parametric tests, namely which one of the two tests was used. In addition, include information if a statistical software was used.

**Information were included in the statistical analysis section.**

 6.Results = Please, clarify “substantively”. Also, depict the results for bodyfat.

**Substantive significance is specified in the statistical analysis section.**

 7.Discussion = cite past research findings related to the outcome of this study and the mechanisms involved, especially in 6th and 7th paragraph. In addition, add in the paragraph with the limitations of the study the small sample size and the lack to establish intersexual differences. Finally, add a final paragraph with the strengths of the study and the recommendations for future research.

**Limitations were added**

 8.Conclusion = add a couple of practical implications for coaches and practitioners.

**We included following practical implications for coaches and practitioners.**

**- Heart rate dynamics during exercise at altitude is higher compare at low altitude: the average HR value at the end of the test at ANE intensity was 6 beats per minute higher at artificially induced altitude than at low altitude.**

**- For the management of endurance sports training at higher altitudes, our research also confirmed that exercise must respect the known principles of acclimatization processes.**

**- The lactate paradox also occurs in a short-term artificially induced hypoxic environment.**
